# Harnessing the Physiological Functions of Cellular Prion Protein in the Kidneys: Applications for Treating Renal Diseases

**DOI:** 10.3390/biom11060784

**Published:** 2021-05-22

**Authors:** Sungtae Yoon, Gyeongyun Go, Yeomin Yoon, Jiho Lim, Gaeun Lee, Sanghun Lee

**Affiliations:** 1 Stembio, Ltd., Entrepreneur 306, Soonchunhyang-ro 22, Sinchang-myeon, Asan 31538, Korea; yoon.st@yahoo.com; 2 Department of Biochemistry, Soonchunhyang University College of Medicine, Cheonan 31151, Korea; ggy0227@naver.com (G.G.); lun4905@naver.com (G.L.); 3 Department of Biochemistry, BK21FOUR Project2, College of Medicine, Soonchunhyang, Cheonan 31151, Korea; 4 Medical Science Research Institute, Soonchunhyang University Seoul Hospital, Seoul 04401, Korea; yoonboo15@naver.com (Y.Y.); wlenfl1@naver.com (J.L.)

**Keywords:** cellular prion protein, PrP^C^, PRNP, kidney, chronic kidney disease, renal cancer, mesenchymal stem cell, renal injury, renal fibrosis

## Abstract

A cellular prion protein (PrP^C^) is a ubiquitous cell surface glycoprotein, and its physiological functions have been receiving increased attention. Endogenous PrP^C^ is present in various kidney tissues and undergoes glomerular filtration. In prion diseases, abnormal prion proteins are found to accumulate in renal tissues and filtered into urine. Urinary prion protein could serve as a diagnostic biomarker. PrP^C^ plays a role in cellular signaling pathways, reno-protective effects, and kidney iron uptake. PrP^C^ signaling affects mitochondrial function via the ERK pathway and is affected by the regulatory influence of microRNAs, small molecules, and signaling proteins. Targeting PrP^C^ in acute and chronic kidney disease could help improve iron homeostasis, ameliorate damage from ischemia/reperfusion injury, and enhance the efficacy of mesenchymal stem/stromal cell or extracellular vesicle-based therapeutic strategies. PrP^C^ may also be under the influence of BMP/Smad signaling and affect the progression of TGF-β-related renal fibrosis. PrP^C^ conveys TNF-α resistance in some renal cancers, and therefore, the coadministration of anti-PrP^C^ antibodies improves chemotherapy. PrP^C^ can be used to design antibody–drug conjugates, aptamer–drug conjugates, and customized tissue inhibitors of metalloproteinases to suppress cancer. With preclinical studies demonstrating promising results, further research on PrP^C^ in the kidney may lead to innovative PrP^C^-based therapeutic strategies for renal disease.

## 1. General Characteristics of Cellular Prion Proteins in Kidneys

A cellular prion protein (PrP^C^) is a glycoprotein on the cell surface. This particular protein has drawn extensive attention and investigation since it was first proposed that infective misfolded PrP^C^ could be responsible for various neurodegenerative disorders that are often referred to as transmissible spongiform encephalopathies (TSEs) or, more commonly, prion disease [1,2]. There is a growing body of evidence that suggests that cellular prion proteins are involved in various cellular processes, and alterations in PrP^C^ expression can result in significant changes in cellular physiology [3,4,5]. PrP^C^ is found in various organs and systems [6], but detailed characterization of the extra-neuronal roles of PrP^C^ has trailed behind [7]. In this review, we provide an overview of the functions of PrP^C^ in the kidneys and examine the experimental strategies involving PrP^C^ to treat kidney diseases.

**Figure 1:**
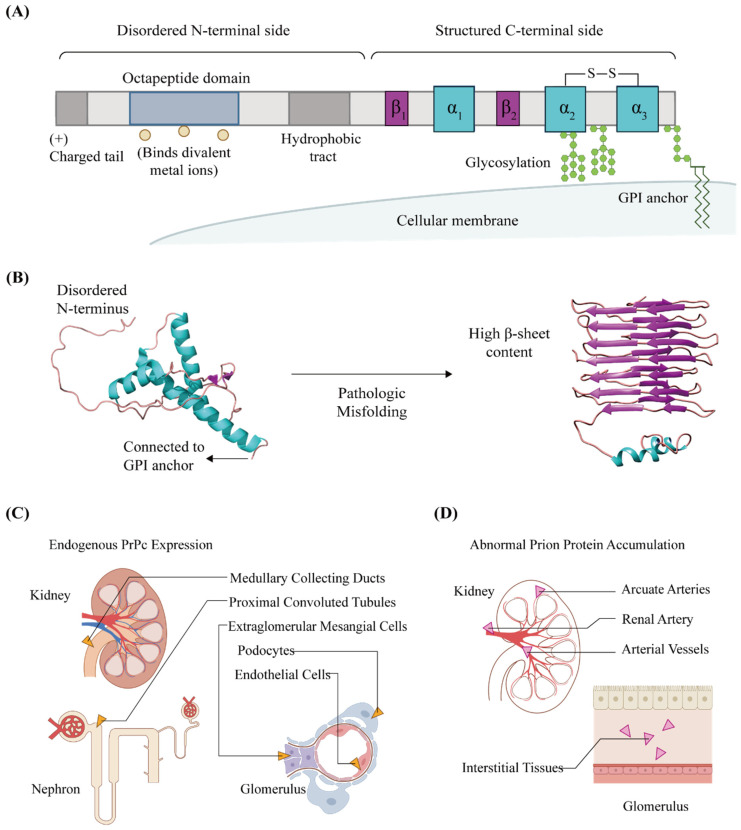
Schematics for the general PrP^C^ protein structure and expression profile of PrP^C^ in kidney tissues. (**A**) A cellular prion protein has multiple distinctive domains. One side of the protein has a helical structure with a GPI anchor, while the other side is an intrinsically disordered polypeptide chain. An octapeptide domain binds to divalent metal ions. (**B**) Ribbon diagram of the human cellular prion protein (PBD 5yj5) is shown on the left. Pathogenic misfolding of a cellular prion protein can lead to an alternate structure with a high beta sheet content. This is shown on the right with the visualization of PrP^Sc^ based on the 4RβS architecture, as proposed by Spagnolli et al., which is one of the more accepted provisionary models for the currently ill-defined PrP^Sc^ structure. (**C**) Endogenous expressions of a cellular prion protein were found in nephron and glomerulus structures. (**D**) Accumulation of an abnormal prion protein in kidney tissues is observed in various prion disease, where the kidney functions are negatively affected. Prion proteins are taken up by a kidney for filtration and excreted into the urine.

It has been well-documented that PrP^C^ exists in two distinct conformations: first, the host-encoded cellular prion protein that carries out the normal physiological functions (PrP^C^), and second, the misfolded infective isoform that is often called pathogenic prion protein (usually denoted as PrP^Sc^) [7]. PrP^C^ is encoded by the *PRNP* gene located on chromosome 20 in humans and chromosome 2 in mice (*Prnp*) [8]. Translation of the *PRNP* gene results in the production of PrP^C^, which undergoes post-translational modification to reach the mature polypeptide comprised of 208 amino acids (Figure 1A). PrP^C^ is anchored to the outer leaflet of the cellular membrane through its connection to glycosylphosphatidylinositol (GPI). Two short antiparallel beta sheet strands and three alpha helices of the C-terminus contribute to the overall structure of PrP^C^, while its pathogenic misfolding (or PrP^C^-to-PrP^Sc^ conversion) could lead to an alternative conformation with a high beta sheet content (Figure 1B). It is important to note that the representation of the PrP^Sc^ structure in this article corresponds to the 4-rung beta-solenoid (4RβS) model of PrP^Sc^ [9], which is one of the more accepted provisory models for illustrating the prion protein structure, as no definitive answers have been given to the structure of PrP^Sc^. There is an ongoing debate on the existence of the third beta sheet (not shown in our illustration) in the PrP^C^ structure, which may have role in PrP^C^-to-PrP^Sc^ conversion [3]. Interestingly, PrP^C^ may undergo further modifications with proteolytic processing at the central region, the C-terminus, or GPI anchor to achieve several different isoforms of PrP^C^ [10].

PrP^C^ is taken up and cleared by the kidney [11]. Two in situ hybridization studies found high levels of PrP^C^ mRNA in kidneys [12,13], while a different article reported a moderate PrP^C^ mRNA expression [14]. PrP^C^ is abundantly expressed and selectively present in podocytes, which constitutes the epithelial lining around glomerular capillaries and their neighboring extraglomerular mesangial cells in the Bowman’s capsule [15]. Endogenous PrP^C^ are present in the proximal convoluted tubules, medullary collecting ducts, renal extraglomerular mesangial cells (EMC), podocytes, and endothelial cells (Figure 1C), as confirmed with mapping of the PrP^C^ protein expression through immunohistochemistry and the quantification of PrP^C^ via Western blotting [16]. Interestingly, the level of PrP^C^ in the kidney seems to vary depending on the age of the subject animal [15] and tissue type [17,18]. In a similar vein, there seems to be a temporal aspect to changes in the PrP^C^ mRNA levels [19,20], and the isoform profile of PrP^C^ was observed to be tissue-specific [14,21]. These observations suggest that delineating the functions of PrP^C^ may require cross-validation across different tissue types.

Abnormal prion proteins (or prion) are detected in the kidneys affected by prion disease (Figure 1D). Creutzfeldt-Jakob disease (CJD) is a fatal prion disease that presents with rapidly progressive dementia [22], and prion proteins were found in the kidneys and urine of sporadic CJD (sCJD) patients [23]. Protease-resistant variants of the prion protein were found in the kidneys of patients affected by variant CJD (vCJD) [24]. In chronic wasting disease (CWD), another prion disease affecting cervids, the immunolabeling of infective prion proteins consistently stains arterial vessels, the wall of the main renal artery, arcuate arteries at the junction of the renal cortex and renal medulla, and renal glomeruli [25]. Scrapie-affected sheep have pathological prion proteins (PrP^Sc^) deposited at the intraepithelial and interstitial tissues of the kidney [26]. Some of these prion proteins detected in the kidneys arrive through the blood circulation for glomerular filtration [11], while others are generated by the kidneys themselves [14]. Prion proteins are filtered into urine [27], and urinary prion proteins could be a source of prion disease infection [28,29] or serve as a biomarker for a noninvasive diagnosis of prion disease [30]. For example, in sCJD patients, prion seeding activities occurs in the kidney as a result of infective prions flowing out from the central nervous system and infecting kidneys and adrenal glands to produce pathological prion proteins onsite [31]. The detection of these disease-related prion proteins via urine screening is suggested as a novel method for diagnosing sCJD [32].

**Figure 2:**
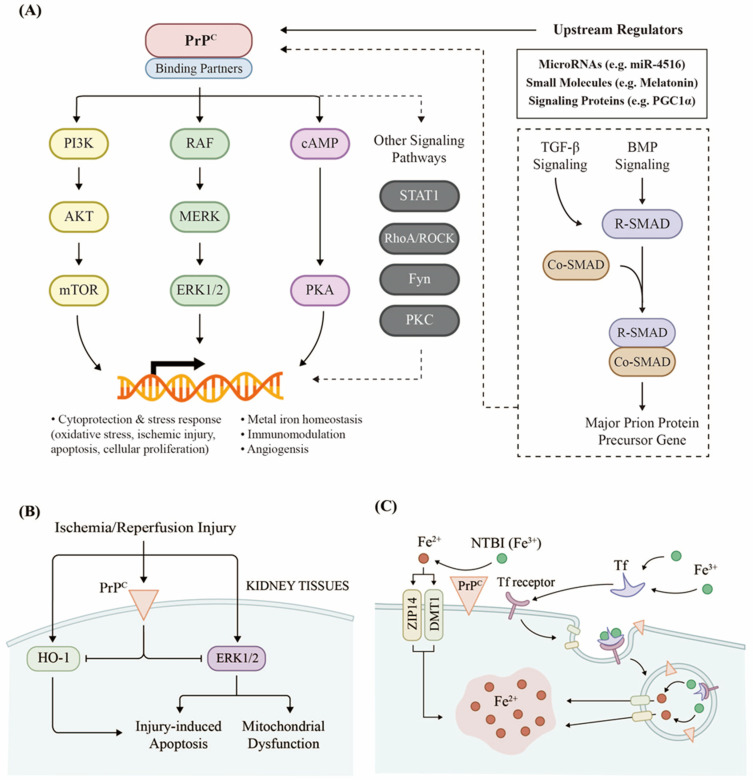
Endogenous PrP^C^ in the kidneys plays a role in cellular signaling processes to influence renal physiology. (**A**) PrP^C^ mediates a variety of downstream signaling pathways via interactions with appropriate binding partners in kidney tissues. There are numerous PrP^C^-signaling pathways that are characterized in neuronal cells, and some of these have been cross-validated in the kidney, while others have not. The signaling proteins and interaction mechanisms that merit further validation in renal models are shown in grayscale or dotted lines. (**B**) PrP^C^ has been associated with some protective functions against ischemic injury through affecting the ERK1/2 transduction pathway and heme oxygenase-1 (HO-1). (**C**) PrP^C^ exhibits ferrireductase activity to promote the uptake of transferrin- (Tf) and non-Tf-bound iron (NTBI) by the kidneys. Fe^3+^ ions are bound to transferrin, which can enter proximal tubular cells. PrP^C^ ferrireductase activity produces Fe^2+^, which can be released into the cytoplasm through DMT1 and ZIP14.

The general functions of PrP^C^ have been extensively investigated via gene knockout experiments. The putative functions associated with PrP^C^ (Figure 2A) include the stress response [33,34,35,36], providing protective effects against oxidative stress [37,38,39], cellular differentiation [40,41,42], neuronal excitability [43,44,45], myelin maintenance [46,47,48], circadian rhythm [49,50], metal ion homeostasis [51,52,53], modulation of the immune system [54], the regulation of amyloid beta and tau protein [55], control of the cellular signaling pathways [56,57], and a few other common cellular processes [7]. Since PrP^C^ downregulation occurs during the disease incubation period [58], the loss of normal PrP^C^ function may partly underlie the pathology of prion protein-related diseases, and PrP^C^ could be targeted for therapeutic purposes. There is evidence for the PrP^C^-mediated suppression of apoptotic signals [59] via TNF [60], BAX [61], or caspase-dependent [62] pathways. The PrP^C^ level is altered in ischemic and hypoxic injury [63], and the severity of the ischemic injury was reduced with the adenovirus-mediated promotion of PrP^C^ [64] but aggravated when PrP^C^ was absent [65].

With the expanding understanding of normal PrP^C^ functions, renal PrP^C^ is becoming an increasingly relevant factor for renal physiology and pathophysiology. In the past, PrP^C^ in kidney tissues has been only used as a proxy model for studying neuronal PrP^C^ [66], which led to the current unavailability of literature dedicated towards examining the functions of PrP^C^ in the kidneys. Given that recent studies on kidney injuries and PrP^C^ have demonstrated that PrP^C^ could be a reliable biomarker for renal injury [67] and protect cells against a chronic kidney disease (CKD) environment [68], further exploration of the link between various kidney diseases and endogenous PrP^C^ is greatly merited. Throughout this review, we have summarized the current understandings of PrP^C^ in the kidneys and its application in renal pathologies and identified potential PrP^C^-based therapeutic strategies for major kidney diseases, including acute and chronic kidney injury, renal fibrosis, and cancers.

## 2. Physiological Functions of PrP^C^ in the Kidneys

Over the course of the past couple of decades, endogenous PrP^C^ has been linked to a wide array of cellular processes with many interacting partners [7], but not many of the putative functions of PrP^C^ with respect to neuronal tissues have been tested or confirmed specifically for their relevance in renal physiology. Nevertheless, the similarities between neurons and certain kidney cells allows for the speculation that some of the endogenous PrP^C^ functions observed in neurons may also remain present in the kidney environment [66]. For example, renal glomerular podocytes have major processes that share important cellular characteristics with neuronal dendrites, and these two types of cells share similar expressions of various molecules involved in signal transduction, trans-membranous transport, and intercellular contacts [69].

The synaptic-like mechanism of communication among glomerular cells also significantly resembles neurons [70], and some iconic neuronal proteins are not only expressed but also play a significant role in the formation and function of renal podocyte processes [71]. The recognition of these similarities motivated the further studying of cellular prion proteins in the kidneys, which led to the discovery that PrP^C^ is present in many kidney tissues, including podocytes [15,16], as discussed previously. Further investigation is needed to verify whether the aforementioned similarities help cellular prion proteins carry out in the kidneys some of the functions PrP^C^ exhibit in neurons. In this section, we highlighted some of the functions of PrP^C^ as they are supported by the currently available in vitro and in vivo data related to the kidneys.

### 2.1. PrP^C^ Regulates Renal Cellular Signaling

A combination of PrP^C^ and its diverse interacting partners can initiate a variety of downstream cellular signaling pathways (Figure 2A). Several review articles have gathered experimental evidence across different experimental models for the PrP^C^-mediated regulation of the ERK1/2 [72,73,74], PI3K/Akt [74,75,76,77], cAMP/PKA [72,78], Fyn kinase (Src family kinase) [79,80], RhoA/ROCK [81], and PKC [74] transduction pathways. It is noteworthy that, except for the ubiquitous effectors, many PrP^C^-mediated signaling pathways have neurospecificity [82], and the complex crosstalk networks between the major signal transduction pathways pose a significant challenge for identifying the direct effectors of PrP^C^. In addition, the context-dependent regulation of signal transduction implies that many of these PrP^C^-mediated pathways may result in different or even contradictory outcomes in different tissues or cell types [7]. Therefore, we focused on the documented cases of PrP^C^-mediated regulation of cellular signaling as they are verified in renal cells or kidney tissues.

Extracellular signal-regulated kinases 1/2 (ERK1/2) are some of the best characterized kinases within the mitogen-activated protein kinase (MAPK) family, and ERK1/2 are activated via phosphorylation (pERK) after the incidence of ischemia/reperfusion injury [83,84] to exercise a protective effect [85,86]. In this case, PrP^C^ prevents the cellular damage caused by an overdrive of ERK activity [87,88]. A more detailed look at the temporal changes in the pERK levels in PrP^C^ knockout and wild-type mice upon renal injury led to interesting observations where the pERK levels continually and steadily increased from day 1 to day 3, while the pERK levels in PrP^C^-null mice showed a sudden spike on day 1, followed by a significant decline far below the wild-type level on day 2 and another more reasonable increase on day 3 [86]. The spike in pERK in PrP^C^ knockout mice was observed mostly in the renal tubular cells, which means that the ERK pathway drove the renal tubular damage in the absence of PrP^C^ [86]. These intriguing patterns of the PrP^C^ deletion-associated pERK phenotype in kidney damage warrant further study, especially considering the strong potential of crosstalk between the different signaling modules. Other literature suggests the involvement of PrP^C^ in the STAT1 pathway, AKT pathway, and caspase-3-mediated pathways [65,87], but these have not been directly verified for kidney tissues.

In addition, PrP^C^ may act as the downstream effector of growth factor signaling in the kidneys. Proximal tubule epithelial cells (PTEC) undergo necrosis under renal ischemic insult, and the damage in the tubules and interstitium of the kidneys can be measured to assess the kidney function and renal health [89]. PTECs trigger an inflammatory response due to kidney injury by producing proinflammatory chemokines and cytokines, which could aggravate ischemic kidney disease [90]. Bone morphogenic protein-7 (BMP-7), which is part of the TGF-β superfamily, is known to play an important role in epithelial tubule development [91], and the expression of BMP-7 in adult kidneys was related to the decrease in the inflammatory response in kidney injuries [92,93,94]. In a rodent model, the direct administration of BMP-7 to the kidneys with renal ischemia led to the increased survival and decreased inflammatory damage of kidney tissues [95,96]. A gene array analysis used to identify the genes regulated by BMP-7 in primary human proximal tubule cells revealed that the major prion protein precursor gene showed an almost two-fold decrease in response to the BMP-7 treatment in the presence of proinflammatory cytokine TNF-α [97]. Although the protein-level expression of PrP^C^ needs further validation, this data provides evidence that additional research may identify many other signaling pathways to either regulate or be modulated by PrP^C^ in renal tissues.

Furthermore, the PrP^C^-dependent cellular signaling pathways could be modulated with certain small molecules and nucleic acids that act as upstream regulators of PrP^C^. Tauroursodeoxycholic acid (TUDCA) was found to induce the Akt-dependent PrP^C^ signaling cascade to reduce ER stress-related cell death and to improve angiogenesis [98], and a treatment with pioglitazone inhibited apoptosis, ameliorated dysregulation in mitochondrial fusion and fission, and improved mitophagy via the PGC-1α/PrP^C^ axis [99]. In addition, a melatonin treatment was found to promote miR-4516 to upregulate PrP^C^ to improve the mitochondrial function [68]. The identification of the miR-4516/PrP^C^ interaction in CKD suggests that there may be many other microRNAs acting upstream of PrP^C^ to modulate the cellular processes in the kidneys. Recently, a genome-wide library screening of miRNA mimics reported several high-confidence hits for the regulation of human PrP^C^ [100], and recognition for the importance of renal RNA and miRNA profiles in progressive kidney diseases is on the rise [101,102,103,104]. Different strategies to target PrP^C^-mediated renal cellular signaling with the purpose of designing and developing treatments for kidney disease are discussed in greater detail in later sections of this review.

### 2.2. PrP^C^ Responds to Kidney Injury

The cellular prion protein has been associated with neuro-protective functions, and among them, protection against ischemic brain injury is one of the best-characterized roles of PrP^C^ [85]. In the brain, hypoxic damage triggers PrP^C^ mRNA upregulation and PrP^C^ accumulation in a murine model [63] and human patients [105]. PrP^C^ deficiency leads to greater infarction [63] through the ERK and STAT1 pathways [87] or Akt activation and post-ischemic caspase-3 activation [65], and the overexpressing PrP^C^ via adenovirus-mediated gene targeting demonstrated reduced ischemic injury [64]. This is another PrP^C^ function that is suspected to have broad applicability in other peripheral organs that are susceptible to ischemic injury.

In fact, when the murine PrP^C^ gene was overexpressed in rabbit kidney epithelial cells and treated with a hydroxyl radical-generating chemical toxin (paraquat), the PrP^C^-overexpressed cells were found to have a significantly reduced chemical-induced cell toxicity, DNA damage, and lipid peroxidation, while demonstrating enhanced superoxide dismutase and glutathione peroxidase activities [106]. Subsequently, this protective role of PrP^C^ was later confirmed in the heart [38], and this inspired a similar investigation for the kidney. Zhang et al. found that the PrP^C^ levels were significantly increased upon ischemia/reperfusion injury (IR injury) in mice kidneys compared to the control kidney from healthy mice, and IR injury in PrP^C^ knockout mice resulted in more severe tubular damage, worse renal dysfunction, increased oxidative stress markers, impaired mitochondrial respiratory chain functionality due to decreased expressions of complexes I and III, and enhanced phosphorylation of the ERK pathway compared to the wild-type control [86].

Taken together, there seems to be sufficient evidence for PrP^C^-mediated protection against renal IR injury, particularly through modulation of the mitochondrial function and the ERK signaling pathway (Figure 2B). This provides a plausible explanation for why PrP^C^ is secreted by renal epithelial cells under endoplasmic reticulum stress [67]. The current efforts to take advantage of such protective functions of PrP^C^ will be reviewed in the following sections.

### 2.3. PrP^C^ Promotes Iron Uptake in the Kidneys

One of the few well-documented physiological functions of PrP^C^ in the kidneys is its role in iron uptake (Figure 2C). Interestingly, the documentation of this effect was motivated by the convenience of using the kidney as a proxy model to study the dysregulation of iron homeostasis in the central nervous system. The alteration of iron homeostasis by the loss of PrP^C^-mediated conversion of Fe^3+^ reduction to Fe^2+^ has been speculated as an important cause of neurotoxicity in prion disorders [107], but a more detailed characterization of this model was carried out using in vivo and in vitro kidney models, where a PrP^C^-promoted uptake of transferrin- (Tf) and non-Tf-bound iron (NTBI) via ferrireductase activity in the kidneys was confirmed [66,108]. A ferrireductase-deficient mutant of PrP (PrPΔ51–89) lacked this activity [66], suggesting that PrP^C^ promotes the retrieval of iron via its ferrireductase activity. Additional research unveiled the mechanism of such PrP^C^ modulation of kidney iron metabolism: a cellular prion protein acts as a ferrireductase partner and regulator for divalent metal iron transporters ZIP14 and DMT1 [109,110].

PrP^C^ modulation of the iron uptake is an example of how understanding of the PrP^C^ function was improved through expanding the scope of a PrP^C^ study beyond neuronal tissues. These results not only provided valuable data for studying neurodegenerative disease but also had more immediate applications for kidney disease. Iron homeostasis is critical for multiple physiological processes, and an imbalance in iron homeostasis has been associated with multiple pathological processes [111]. As the kidney plays a vital role in iron homeostasis through the renal reabsorption of iron ions [112,113], acquired or inherited disturbances in this process can cause systemic or local iron accumulation or iron deficiency, both of which lead to adverse pathological outcomes [114,115,116]. Therefore, targeting the iron homeostasis pathway may serve as a therapeutic target [117] to either prevent or delay progressive kidney diseases [118].

## 3. PrP^C^ and Kidney Disease

The normal function and characteristics of PrP^C^ functions, as mentioned in the previous sections, are implicated in some of the most common kidney diseases that are not classified as prion disease. The application of these properties to develop PrP^C^-based therapies may lead to innovative approaches that could compensate or overcome the limitations of the currently available treatment options for many kidney diseases, including acute kidney injury, chronic kidney disease, renal fibrosis, and renal cancer (Table 1). There are other kidney diseases like IgA nephropathy where the implication of PrP^C^ processes have been suggested [119] but understanding of the underlying mechanism of endogenous PrP^C^ involvement is absent. Therefore, taking advantage of the properties of PrP^C^ to achieve clinical success is dependent on elucidating the exact roles of PrP^C^ in the target disease.

Here, it could be appropriate to reiterate that the focus of this section is on the kidney diseases that may have pathogenic mechanisms involving alterations in the expression or functions of cellular prion proteins, rather than prion disease affecting kidney functions through the accumulation of abnormal prion proteins. The former does not generate infectious proteins, and PrP^C^ is implicated as a modulator of important cellular processes relevant for the disease etiology [142]. Therefore, the altered PrP^C^ levels we discuss in relation to chronic kidney disease are mostly unrelated to the transmissible misfolded PrP^Sc^ that is critical in prion disease. 

### 3.1. PrP^C^ and AKI/CKD

The kidneys play a critical role in maintaining the overall health of an individual by filtering toxic metabolic waste products [122] and maintaining adaptive tissue repair programs [67,143]. Compromised kidney functions often have multifactorial etiology [120,144,145,146,147,148,149], but they all progress towards chronic kidney disease (CKD), which is often diagnosed by a decreased glomerular filtration rate and persistently elevated levels of serum creatinine and albuminuria [143]. Acute kidney injury (AKI) has been formerly known as acute renal failure that affects many hospitalized patients and critically ill patients and is characterized by a rapid loss of the kidney’s excretory function, measured by the accumulation of nitrogen metabolism end products (urea and creatinine) [150]. Chronic kidney disease (CKD) has been defined by a reduced glomerular filtration rate and increased urinary albumin excretion for three months or longer [151]. AKI is very common among hospitalized patients, with mortality above 50% for critically ill patients [150]. CKD is affecting an increasingly higher number of people worldwide, with the global population aging very quickly, and CKD patients often led to premature deaths and a loss of disability-adjusted life years [151]. Renal replacement therapy is indicated for AKI and CKD without any effective cure [152,153].

Both AKI and CKD are heterogenous diseases with varying pathogenic mechanisms, and there is strong evidence suggesting that they are closely related, as CKD can both be a predisposition for AKI or result from recurrent or sustained AKI [154,155,156]. One of the most significant pathologic processes of kidney damage is renal ischemia/reperfusion injury (IRI), which is a common cause for AKI, and the subsequent transition into CKD [121,157,158,159,160]. As PrP^C^ protects against renal IRI via its engagement with the ERK1/2 pathway [86], promoting this effect in the kidneys may lead to therapeutic outcomes. In fact, an overexpression of PrP^C^ through adenovirus-mediated gene transfer reduced cerebral ischemic injury and improved neurological dysfunctions in rats [64,88]. Investigation into similar approaches for renal IRI is merited.

Another important factor in AKI and CKD is the iron level. Iron is known to be essential for the health and normal functioning of many tissues, including the kidneys but, specifically for CKD and AKI, iron dysregulation initiates of oxidative stress, mitochondrial dysfunction, and inflammation [118]. Glomerular and renal tubular cell injuries may increase the iron content in an intracellular space, and reducing these excessive luminal or intracellular iron levels in the kidneys has been proposed as a promising approach to treat AKI and CKD [115]. Since a prion protein (PrP^C^) functions as a ferrireductase at the apical side of the proximal tubule epithelial cells, and renal iron handling mechanisms differ from one nephron segment to another [66], we may speculate that some subset of AKI or CKD etiology may be based on disturbances in the PrP^C^ function, and the identification of these subtypes may be helpful in delineating different causes of kidney failure to find better intervention strategies.

On a different note, PrP^C^ may be useful for improving the therapeutic efficacy of autologous cell therapy for CKD. As the regenerative ability of the kidneys after injury is quite limited, the ability of MSCs to trigger regenerative processes, through paracrine activities [161] after migration to the injury site via the secretion of various growth factors and cytokines or extracellular vesicles filled with cell-to-cell signaling factors like microRNA, is of great interest for developing AKI or CKD treatments [162]. Recently, a systemic review and meta-analysis of preclinical studies on applying cell-based therapy for chronic kidney disease concluded that the use of mesenchymal stem/stromal cells (MSCs) was associated with the highest efficacy [163]. The injection of autologous adipose tissue-derived MSCs for the treatment of CKD was reported as clinically safe in a pilot study [164]. There have been quite a few clinical trials that have reached phase I or Phase II for MSC-based cell therapy for different types of CKDs, though no large-scale trial has been reported due to the difficulty associated with the mass production of qualified MSCs [165,166].

Despite the potential of MSCs for realizing personalized medicine, the viability of MSCs, and their capacity to induce regenerative processes, is greatly diminished when they are faced with an adverse renal microenvironment (including a hypoxic environment, proinflammatory condition, free radical-induced oxidative stress, etc.) at damaged tissues of diseased kidneys [167]. Therefore, enhancing the functionality and therapeutic efficacy of MCS-based cell therapy depends on providing adequate protection against the hostile microenvironment of injured kidneys for AKI and CKD patients [166]. There have been a lot of progress in elucidating the interaction between MSC and the microenvironment of the engraftment site, and this provides a new opportunity to engineer a therapeutic effect [127,168,169]. Controlling the growth condition is one common way to improve the functions of MSCs [127,166], and PrP^C^ is a promising target for enhancing their therapeutic efficacy [170]. Some of the experimentally validated interventions to alter the growth conditions of MSCs to improve the PrP^C^-mediated protective effects are summarized below.

First, tauroursodeoxycholic acid (TUDCA), or bile acid, has been found to reduce the proteins associated with the damage from ischemia-induced endoplasmic reticulum (ER) stress in MSCs [98,171]. Investigation into the mechanism has revealed that TUDCA effectively protects MSCs through an Akt-dependent PrP^C^-signaling cascade in vivo and in vitro, while effectively reducing ER stress-related cell death and improving angiogenesis [98]. TUDCA–PrP^C^ protection of MSCs has been verified with a P-cresol-induced CKD mice model. In the presence of the uremic toxin P-cresol, ROS-mediated ER stress increased cell death in SH-SY5Y cells, and a coculture with TUDCA-treated CKD-hMSCs increased the antioxidant enzyme activities in SH-SY5Y cells through upregulation of the PrP^C^ expression, which was responsible for the observed protective effects against CKD-mediated ER stress and apoptosis [172].

Second, PrP^C^ responds to treatment with melatonin, a pineal gland secretory hormone associated with the regulation of circadian rhythms and homeostasis [173]. When MSCs are pretreated with melatonin, they show an improved survival rate at the damaged tissues and demonstrate an increased capacity for improving angiogenesis, renal cell proliferation, and overall renal function, as measured by the decrease in plasma creatinine and urea [174]. Mitochondrial function was also improved in a PrP^C^-dependent manner in the CKD mice model with a melatonin pretreatment [175]. Instead of using melatonin directly on MSCs derived from CKD patients (CKD-MSCs), healthy MSCs can be utilized to produce exosomes with reno-protective signaling molecules. Treating healthy MSCs with melatonin increased the PrP^C^ in exosomes isolated from MSCs through the upregulation of miR-4516 and, when CKD-MSCs are treated with these exosomes from melatonin-treated healthy MSCs, a significant increase in the levels of angiogenesis-associated proteins through miR-4516-PrP^C^ signaling [68]. This approach seems to mitigate the challenge with the innate functional impairments of CKD-MSCs due to their adverse growth conditions caused by exposure to uremic toxins circulating in the body of CKD patients.

In this context, miR-4516 and PrP^C^ augment the regenerative potential of MSCs and MSC-derived extracellular vehicles (EVs) for AKI or CKD treatments. This data is very informative for developing a therapeutic application of EVs for kidney disease. EVs are nanosized vesicles released by various cells and are known to participate in inter-nephron cellular communication [176,177]. The application of EVs as a therapeutic vector is becoming increasingly popular for renal disease [178,179,180], yet a wide variety of different combinations of source cells and effective molecules are still being tested to search for the best treatment regimen for CKD [181]. Mesenchymal stem cells [182,183], endothelial progenitor cells [184], and tubular epithelial cells [185] are some of the leading contenders for EV biogenesis, while using EVs loaded with either heterogeneous natural contents [186], small immunoregulatory proteins [187], or microRNAs [68,188,189], as effective molecules are yielding promising results in AKI- or CKD-related preclinical studies [181]. It is plausible that miR-4516 or PrP^C^ could be employed as potent effective molecules to enhance EV-based therapies for kidney injuries.

Lastly, pioglitazone, an antidiabetic medication used to treat type 2 diabetes, also increases the expression of cellular prion proteins (PrP^C^) in CKD-MSCs. In one study, a pioglitazone treatment increased the expression levels of proliferator-activated receptor gamma coactivator 1-alpha (PGC-1α), which, in turn, upregulated the PrP^C^ expression in CKD-MSCs. The activation of the PGC-1α/PrP^C^ axis inhibited apoptosis, ameliorated dysregulation in mitochondrial fusion and fission, and improved mitophagy [99]. In a different study, this PrP^C^-mediated regulation of mitophagy was associated with the upregulation of phosphatase and tensin homolog (PTEN)-induced putative kinase 1 (PINK-1) via nuclear factor κ-light-chain-enhancer of activated B cells (NF-κB) [190]. These data suggest that a pioglitazone treatment could be another way to improve the candidacy for MSCs for the cell-based treatment of kidney injuries.

As mentioned before, the infliction of a kidney injury in either AKI or CKD seems to alter the expression of endogenous PrP^C^ in the kidneys. One study reported that the blood plasma obtained from 20 patients with renal failure showed higher levels of PrP^C^, which was not removed via hemodialysis [191]. However, an examination of the serum sample obtained from 37 CKD patients revealed that PrP^C^ was significantly decreased in the CKD group [68]. This is consistent with our other subsequent studies of PrP^C^ expression in the cells and tissues obtained from CKD patients [68,99,174]. This disagreement may be attributed to a number of factors. First, CKD patients often receive heparin during dialysis, which may impact the PrP^C^ expression level [191]. The difference between the plasma and serum may have played a minor role as well. In addition, it has been suggested that the PrP^C^ response observed in renal failure is not generalizable for different chronic illnesses or an acute phase reaction [191]. This suggests that the PrP^C^ expression in AKI or CKD may vary from one subtype to another, since CKD and AKI are defined by their kidney function, not by the pathogenic factors responsible for renal failure. For example, CKD resulting from von Willebrand disease [192] have very different etiology from CKD with diabetes mellitus [120,123,124], so screening for CKD patients based on the glomerular filtration rate and high creatinine may overlook this confounding variable in characterizing the association between CKD and PrP^C^ expressions. However, all of the studies examined in this review presented a statistically significant difference for the PrP^C^ expressions between the kidneys of AKI or CKD patients and the healthy control groups. Taken together, we can conclude that PrP^C^ dysregulation is implicated in AKI or CKD, but the detail of PrP^C^ involvement awaits further clarification. 

### 3.2. PrP^C^ and Renal Cancer

There is evidence that PrP^C^ contributes to the resistance against tumor necrosis factor α (TNF-α) apoptosis pathway in renal adenocarcinoma. PrP^C^-expressing renal adenocarcinoma cells (ACHN cells) demonstrated a modest but statistically significant increase in cell viability compared with the control group via the suppression of TNF-α-induced cell death, and the PrP^C^ expression in ACHN led to a higher proliferative index [125]. This is interesting, because there is a large volume of research on how PrP^C^ could mediate the tumorigenic effects and promote cancer proliferation, metastasis, drug resistance, and the cancer stem cell phenotype [34]. 

In our previous work, we confirmed the anticancer effects of the anti-prion antibody in a xenograft model and found that a cotreatment of anticancer drugs with the anti-prion antibody can achieve superior efficacy, with a much lower dosing of chemotherapeutic agents in colorectal cancer (unpublished data). In accordance with our findings, another literature on PrP^C^ antibody treatment against colon cancer reported increased apoptosis via reduced Bcl-2 expression; antiproliferative activity; and enhanced effects of irinotecan, 5-FU, cisplatin, and doxorubicin in combination therapy [193].

Antibody–drug conjugates are another category of antibody-based therapeutics under active investigation for their application in cancer [126,194,195,196,197], and PrP^C^ may serve as a potential target for antibody–drug conjugates [34]. Here, aptamers, which are oligonucleotides or peptides that bind to specific target molecules, can be used in lieu of antibodies to create another targeted drug delivery system called aptamer–drug conjugates, as aptamers have a few distinctive advantages over antibodies for their engineering simplicity, rapid tissue penetration, and low immunogenicity [198,199,200]. We previously synthesized PrP^C^ aptamer (Apt)-conjugated gold nanoparticles (AuNPs) for the targeted delivery of doxorubicin, which induced reactive oxygen species generation in colorectal cancer cells by decreasing the catalase and superoxide dismutase activities [128]. Given these discoveries in various cancer types, the conjugation of drugs to either the PrP^C^ antibody or PrP^C^ aptamer may be an effective way to improve drug delivery for kidney cancers.

While a majority of the efforts to take advantage of PrP^C^ in therapeutic development focuses on the regulatory role of PrP^C^ in cellular processes, the properties of PrP^C^ as a cell surface protein can be exploited to formulate a different approach to treating renal carcinoma. Matrix metalloproteinases (MMPs) are a group of zinc-dependent endopeptidases that carries out the degradation of the extracellular matrix (ECM), and ECM degradation by MMPs enables cancer invasion and metastasis [129]. The tumor necrosis factor α (TNF-α)-converting enzyme (TACE), which is an A disintegrin and metalloproteinase (ADAM), is a multidomain transmembrane protein that functions as proteinases to produce cell surface ligands important for cell growth and proliferation by cleaving their membrane-bound precursors (a phenomenon called the ‘ectodomain shedding’ of ADAM) [130]. Both MMPs and TACE are under the regulation of the tissue inhibitors of metalloproteinases (TIMPs); hence, promoting TIMP can lead to tumor suppression [131]. In a recent study, bioengineered TIMP-1 with enhanced affinities for Membrane type 1-MMP (MT1-MMP) and TACE was fused with the glycosyl-phosphatidyl inositol (GPI) anchor of a cellular prion protein to increase the colocalization of the modified TIMP and its inhibitory targets on the cell surface [132]. The transduction of these modified TIMP fused with the PrP^C^ anchor in renal carcinoma triggered cellular senescence, disrupted MMP-mediated proteolysis of the ECM, and suppressed cell motility and survival in vitro and in vivo [132]. Here, a creative incorporation of PrP^C^ into a therapeutic design led to a novel approach to stop the progression of metastatic renal carcinoma. 

### 3.3. PrP^C^ and Renal Fibrosis

The promising preclinical data for applying novel therapeutic approaches based on PrP^C^ for AKI, CKD, and renal carcinoma, as discussed above, invites the question of whether PrP^C^ can be useful in treating other renal diseases. Does PrP^C^ have a role to play in diabetic kidney disease, renal fibrosis, polycystic kidney disease, glomerulonephritis, and other common renal illnesses? Out of all, the involvement of PrP^C^ seems the most plausible in renal fibrosis, which results from an overdrive of maladaptive renal tissue repair programs [133,134,135], as new evidence supporting the link between PrP^C^ and the regulation of renal fibrosis is emerging.

For example, it is known that TGF-β1 act as the master regulator of renal fibrogenesis through the canonical TG-Fβ/Smad signaling pathways, which induces excessive production of the extracellular matrix (ECM) while suppressing ECM degradation [136]. One of the other members within the TGF-β superfamily, BMP-7, which induces the BMP/Smad signaling pathways, was found to regulate the expression of a major prion protein precursor gene [97]. BMP-7 is heavily expressed in kidney podocytes, distal tubules, and collecting ducts [137], which correspond with the tissues of kidneys with endogenous PrP^C^ expression. The disappearance of antifibrogenic BMP-7 in the early stages of renal disease has been associated with the progression of renal fibrosis [138], and antifibrotic BMP-7 activates smad5 to inhibit smad6, which induces the translocation of TGF-β-activated smad3 to the nucleus [137]. If PrP^C^ turns out to be involved in the antifibrotic BMP-7 pathway, it could be another promising target for antifibrotic therapy to intervene with the profibrotic effects of TGF-β signaling at its downstream effector level. This would be a more realistic approach compared to direct targeting of TGF-β, as the latter is unlikely to lead to a viable therapy due to the broad involvement of TGF-β in many essential cellular processes [97].

In addition, we previously demonstrated that a melatonin treatment can suppress renal fibrosis by upregulating miR-4516 in kidney tissues [139], and this is consistent with the widely reported antifibrotic effects of melatonin [140,141,201,202]. More recently, we showed that the melatonin-mediated upregulation of miR-4516 promotes PrP^C^ expression [68]. If the miR-4516-induced upregulation of PrP^C^ plays a role in the observed antifibrotic effects, this would add to the existing evidence supporting the validity of PrP^C^ as a potential target for treating renal fibrosis.

## 4. Conclusions

Despite the emerging body of literature supporting the wide-ranging applicability of PrP^C^ functions in renal physiology and many pathologies where the kidney is either at the center of the disease etiology or suffers from secondary exposure to cellular toxins resulting from other organs, a rigorous characterization of PrP^C^ in kidneys has been trailing behind, and therefore, several questions remain. First, the PrP^C^ expression profile in kidneys under normal and pathologic conditions are not fully understood for various kidney tissues. A low volume of research covering the kidney expression of PrP^C^ poses a great challenge for evaluating the accuracy and reproducibility of each literature. The probing endogenous PrP^C^ expression at the transcript and protein levels in different animal models and serum or plasma samples from a human cohort all concluded that PrP^C^ reports statistically significant differential expressions in kidney tissues for renal and prion diseases, yet the direction of changes in the PrP^C^ expressions diverged, as the reported data alternated between overexpression and downregulation, depending on the model and experimental conditions. This strongly suggests that PrP^C^ expression may be highly context-dependent, and the identification of confounding variables behind PrP^C^ regulation would be a necessary prerequisite in harnessing the functions of PrP^C^ for therapeutic purposes.

Another important question remains for the underlying biological mechanisms of the observed physiological functions of PrP^C^: does PrP^C^ directly regulate all the pathways that seem to be affected by the differential expression of PrP^C^? Ccould there be a few core pathways that are the direct primary targets of PrP^C^-mediated regulation, and the rest are secondary effects caused by the activity of the primary targets? Although many signaling pathways are reportedly activated or inhibited by PrP^C^, the existence of extensive crosstalk networks between the pathways, coupled with the differential expression of suspected binding partners of PrP^C^ in different tissues/cell types, point towards the probability that there will be a difference in the degree of involvement for the primary and secondary downstream effectors of PrP^C^ [7]. For example, in the PrP^C^-induced activation of glycolysis in cerebral ischemia, crosstalk between the Wnt/β-catenin and PI3K/Akt signaling pathways is the executor of glycolysis activation, but PrP^C^ interaction with the PI3K/Akt pathway is better documented [7,203]. Mapping the full regulatory network for PrP^C^ in a physiological context could help us identify which cellular pathways are better targeted with PrP^C^-based interventions for specific renal diseases. As the investigation of the PrP^C^ functions is the most advanced in neurological models, testing the cross-applicability of their findings may yield fruitful results.

The translation of a PrP^C^-based treatment paradigm into clinical use in the aforementioned conditions would be a big challenge, not only because of the insufficient understanding of PrP^C^ in renal pathophysiology but also because of the relatively unclear pathologic mechanisms of the disease themselves. Thus, further studies should have a balanced focus on both identifying the role and regulatory network of PrP^C^ and the underlying etiology of renal pathology. Meanwhile, improving the viability and functionality of the MSCs for cell therapy by targeting PrP^C^ in extracellular vesicles seems to be the most promising strategy with the broadest applicability. Undertaking clinical trials to test the PrP^C^-based strategies for enhancing cell therapy for CKD would be the important next step. Taken together, increasing the momentum for researching the cell biology of renal PrP^C^ may provide the necessary foundation for discovering innovative pharmacological or cell-based agents that are effective for kidney failures and renal cancers.

## Figures and Tables

**Table 1:** Applications of the PrP^c^ functions in designing therapeutic strategies for renal diseases.

Kidney Disease	Potential PrP^c^-Based Therapeutic Strategy	Proposed Mechanism for the Roles/Effects of PrP^c^	Validated in Renal Cells	Related References
AKI/CKD	Expressing PrP^c^ in renal tissues to ameliorate IRI renal damage via gene delivery with the adenovirus vector	PrP^c^ suppresses ERK1/2-dependent tissue damage via apoptosis and mitochondrial regulation	Yes	[35,120,121]
	Suppressing PrP^c^ in the kidney to treat the dysregulation of iron homeostasis	PrP^c^ ferrireductase activity promotes DMT1 and ZIP14-mediated iron uptake in kidney tissues	No	[23,119,122]
	Upregulating PrP^c^ in MSC with melatonin, TUDCA (bile acid), or pioglitazone treatment to improve MSC or EV-based cell therapies	PrP^c^ protects MSCs against the adverse microenvironment in damaged kidneys, enhancing the efficacy of the treatment	Yes	[24,123,124,125,126]
Renal Fibrosis	Targeting PrP^c^ downstream of BMP-7 to stop the progression of TGF-β-induced renal fibrosis	PrP^c^ precursor gene is regulated by BMP-7, which disappears in fibrosis. Interventions at the PrP^c^ level could help ameliorate the fibrotic effects	No	[127]
Renal Carcinoma	Cotreatment of the PrP^c^ antibody and chemotherapeutic agents	Reduction of PrP^c^-related drug resistance and metastasis leads to a better outcome	No	[38,128,129]
	Using a PrP^c^ aptamer-conjugated drug delivery system or PrP^c^ antibody–drug conjugates for cancer treatment	Targeting differentially expressed PrP^c^ in cancer cells achieves targeted drug delivery	No	[38,130,131,132,133,134,135,136,137,138]
	Fusion of TIMPs with the GPI anchor domain for cell surface expression	Increased colocalization of PrP^c^-fused TIMP with its inhibitory target (MMP) increases the anticancer effect	No	[139,140,141]
